# Spin-Crossover Materials towards Microwave Radiation Switches

**DOI:** 10.1038/srep38334

**Published:** 2016-12-02

**Authors:** Olesia I. Kucheriv, Viktor V. Oliynyk, Volodymyr V. Zagorodnii, Vilen L. Launets, Il’ya A. Gural’skiy

**Affiliations:** 1 Department of Chemistry, Taras Shevchenko National University of Kyiv, 64 Volodymyrska St., Kyiv 01601, Ukraine; 2 Department of Radio Physics, Electronics and Computer Systems, Taras Shevchenko National University of Kyiv, 64 Volodymyrska St., Kyiv 01601, Ukraine

## Abstract

Microwave electromagnetic radiation that ranges from one meter to one millimetre wavelengths is finding numerous applications for wireless communication, navigation and detection, which makes materials able to tune microwave radiation getting widespread interest. Here we offer a new way to tune GHz frequency radiation by using spin-crossover complexes that are known to change their various physical properties under the influence of diverse external stimuli. As a result of electronic re-configuration process, microwave absorption properties differ for high spin and low spin forms of the complex. The evolution of a microwave absorption spectrum for the switchable compound within the region of thermal transition indicates that the high-spin and the low-spin forms are characterized by a different attenuation of electromagnetic waves. Absorption and reflection coefficients were found to be higher in the high-spin state comparing to the low-spin state. These results reveal a considerable potential for the implementation of spin-crossover materials into different elements of microwave signal switching and wireless communication.

In recent years, a rapid increase in the use of wireless communication tools, digital systems, local area networks, and other equipment, which uses electromagnetic waves in the GHz range, is observed. Meanwhile, an extensive implementation of devices dealing with GHz radiation has provoked serious electromagnetic interference (EMI) and electromagnetic compatibility issues. Consequently, with increasing demand for EMI shielding in both civil and military applications^1^,^2^ a variety of research works have been carried out to investigate microwave absorbers^2^,^3^,^4^,^5^,^6^,^7^,^8^. Such absorbers are beneficial to achieve the attenuation of electromagnetic radiation through the dielectric or magnetic losses, and could be used to minimize the electromagnetic reflection from metal surfaces of ships, aircrafts, tanks and walls of electronic equipment^9^,^10^.

At the same time, developments in microelectromechanical systems (MEMS) technology have made possible the design and fabrication of control devices suitable for switching microwave signals. Micromechanical switches were firstly demonstrated in 1979^11^ by developing electrostatically actuated cantilevers used to switch low-frequency electrical signals. Microwave radiation switches also employ semiconductors^12^, photoconductors^13^ or metamaterials^14^. Today radio frequency switches^15^,^16^ are used to route high frequency signals between different transmission channels or devices, which is an essential component in a wide range of applications including terrestrial and satellite communications, radar systems, and microwave metering equipment.

Switch of an electromagnetic signal can be achieved by using phase transition materials those two forms differently interact with a specific radiation. In this work a class of bistable compounds in perspective of electromagnetic signals control, so called, spin-crossover (SCO) complexes, is analysed. They represent a group of compounds which can undergo spin transition under the influence of such external stimuli as temperature^17^ or pressure^18^, light irradiation^19^, magnetic field^20^ or adsorption of guest molecules^21^. All possibilities of application for these materials are driven by the variation of a whole set of their physical characteristics when the spin state changes. A wide diversity of SCO complexes makes it possible to achieve a material with almost any desirable hysteresis, abruptness and temperature of the transition.

SCO compounds have already shown a huge perspective in the development of MEMS^22^ and electronics elements^23^, that exploit a variation of corresponding mechanic and electric properties of these materials upon spin state change. As photonic materials they attract attention for the development of optical switches^24^, protective elements^25^, microthermometers^26^ etc. A unique sensitivity of their transition properties towards chemical influences make them attractive candidates for the active elements of chemical sensors with optical, spectroscopic, magnetic or other techniques of detection^27^,^28^,^29^.

Properties of SCO complexes were previously investigated in a wide spectral range. A drastic change of absorption induced by spin transition was observed in a range starting from γ-ray to far infrared radiation (Fig. 1). However, despite the huge perspective of its practical application, there was no information about interaction of SCO complexes with microwave radiation.

Here we offer a way of microwave radiation switching by means of SCO process, because along with a drastic change of various physical properties, spin transition is accompanied by a change of important characteristics of a material: permittivity^30^,^31^, permeability, coefficients of reflection^32^ and absorption within the microwave region.

## Results and Discussion

### Spin transition complex

**Figure 2:**
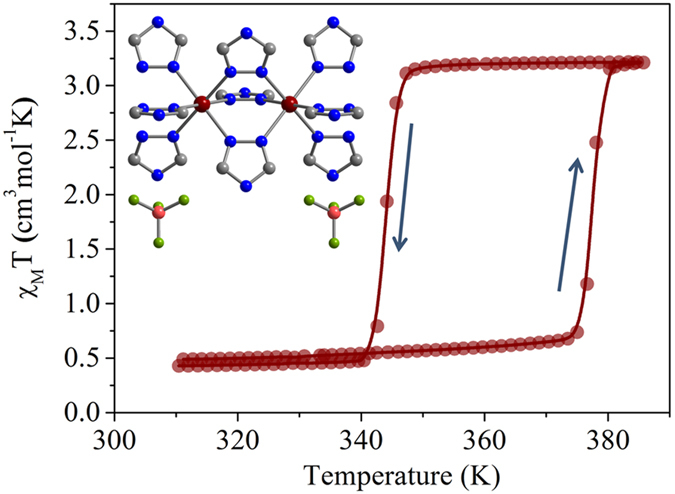
Spin transition in 1. χ_M_T vs. T dependence demonstrates a cooperative transition between diamagnetic and paramagnetic states of the SCO complex (T_1/2_↑ = 377 K, T_1/2_↓ = 344 K). Representation of the structure of **1**^37^ is inserted.

Among all 1,2,4-triazole-based spin crossover compounds, the complex [Fe(Htrz)_2_(trz)](BF_4_)^33^ **1** (Htrz = 1*H*-1,2,4-triazole) is one of the most studied and applied^34^,^35^,^36^. This is because of its uncommon properties that are represented by its abrupt transition, high T_1/2_ and a large hysteresis loop. The thermal spin transition of **1** was demonstrated with SQUID magnetometry (Fig. 2). The change of spin state takes place at 377 K and 344 K in heating and cooling modes respectively. Mössbauer spectrum of **1** measured at room temperature (Figure S1) reveals the doublet that should be attributed to Fe^II^ in the LS state (δ^LS^ = 0.423 mm s^−1^ and ΔE_Q_^LS^ = 0.286 mm s^−1^) at the expanse of residual HS fraction.

### Microwave radiation attenuation

**Figure 3:**
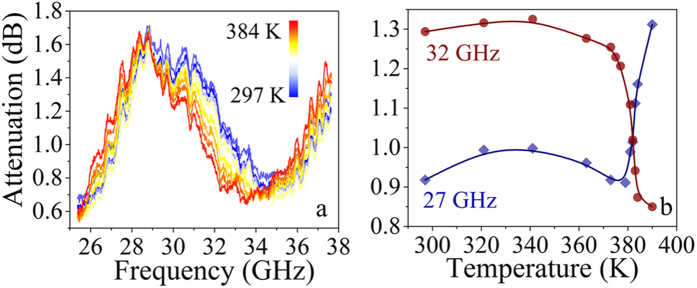
Spin crossover induced change of microwave radiation attenuation. (**a**) Temperature-dependent spectra of microwave radiation attenuation measured in heating mode. (**b**) Dependence of microwave radiation attenuation on temperature at selected frequencies (32 and 27 GHz). Curves demonstrate the effect of a microwave switch associated with SCO.

Temperature dependent attenuation spectra of **1** were acquired using scalar microwave network analyser in 25.8–37.5 GHz region and they are shown in Fig. 3a.

Attenuation in the sample is caused by both absorption and reflection of the electromagnetic wave. At low temperature the minimum of attenuation is found at 34.5 GHz for the LS species. At high temperatures the shift of attenuation frequency minimum towards lower frequencies is observed (33.2 GHz). Such a shift indicates an increase of refraction and permittivity driven by the spin transition.

Temperature dependence of microwave attenuation at selected frequencies (32 and 27 GHz) was measured in heating mode and is given in Fig. 3b. In the temperature range of 295–375 K the attenuation varies just slightly (1.25–1.35 and 0.9–1.0 dB at 32 and 27 GHz, respectively). However, in case of 32 GHz wave analysis in-between 375 and 390 K, the abrupt decrease of the attenuation caused by SCO is observed (transmission at 390 K is 0.85 dB). Inverse effect is observed at the frequency of 27 GHz. This drastic change in the attenuation is related to the spin transition (occurring at 377 K according to the magnetic measurements) whereas the direction of this effect depends on a correlation between the radiation wavelength and the size of the sample.

### Refraction index and absorption measurements

**Figure 4:**
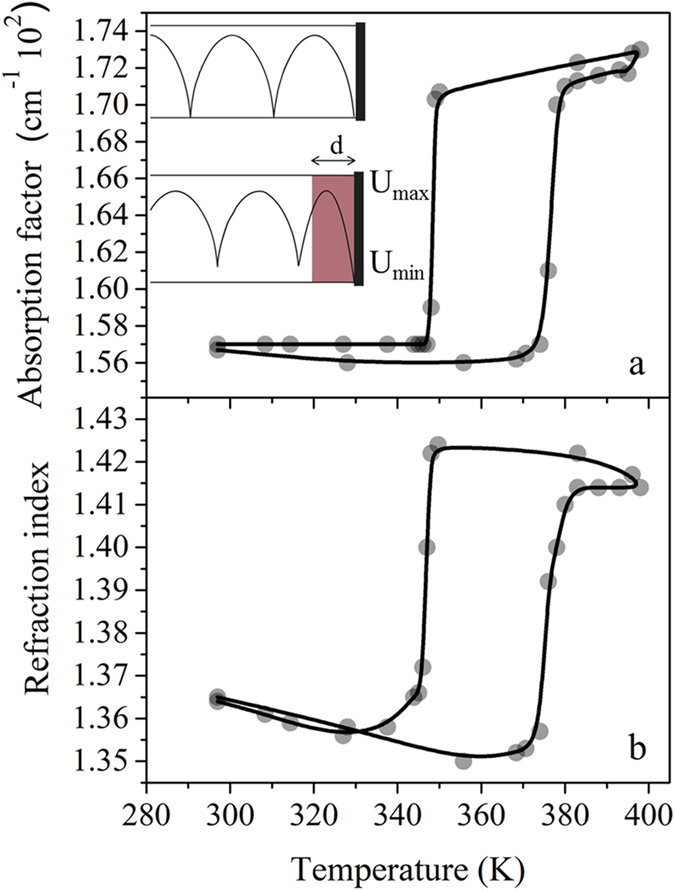
Effect of the spin transition on the microwave properties of 1. (**a**) Temperature dependence of electromagnetic radiation absorption measured at 37 GHz. (**b**) Temperature dependence of refraction index. The curves demonstrate a memory effect in microwave dielectric properties associated with the hysteresis of spin transition measured at 37 GHz. Insert: Schematic representation of a short-circuited waveguide experiment.

To understand the possible microwave absorption mechanism, temperature dependent dielectric properties of the complex were investigated. Shirt-circuit line method, which is a powerful tool to determine dielectric properties of a material, was used to measure SCO induced switch of the refractive index and the absorption factor of **1**. For the implementation of this method, the output of a rectangular hollow metallic waveguide was blocked with a metallic short, inducing short-circuited mode. EM wave in this short-circuited empty waveguide forms a standing wave with a specific amplitude distribution. Coordinates of standing wave’s minima are measured (Fig. 4). The structure of the standing wave in a sample-loaded waveguide differs from the one in an empty waveguide. A measured shift of the minima disposition and an amplitude ratio between minima and maxima of the standing wave gives an opportunity to determine an input impedance of the sample-loaded waveguide^38^. In further calculations the refractive and absorption indexes are extracted.

The absorption factor values were calculated using the input impedance of the loaded waveguide at a given frequency (37 GHz) and the thickness of the sample according to the transmitting line theory, which can be summarized as^38^:


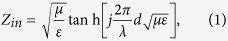



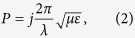



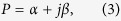



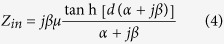


where Z_in_ is the input impedance, β is the phase constant (β = 2π/λ), α is the absorption factor, μ is the permeability (μ/μ_0_ = μ_r_ ≈ 1 for weakly magnetic materials), ε is the permittivity, P is the propagation constant, and d is the thickness of the absorber.

SCO induced shift of the absorption factor is represented in Fig. 4a. One can relate absorption factor to the electromagnetic penetration depth, for which the microwave field magnitude is reduced by the factor 1/e ~ 0.37.

The absorption factor value at low temperatures is equal to ca. 0.0156 cm^−1^ and stays constant up to spin transition temperatures. Along with SCO, an abrupt change of absorption factor is observed (0.0172 cm^−1^ for high spin). Transition temperatures observed in this measurement well corroborate with those found in SQUID experiment.

The values of absorption factor are relatively low compared with those found for triazole complexes in other spectral regions, including THz region^39^, where an absorption coefficient is estimated at 400–600 cm^−1^. This is probably because the investigated frequencies do not correspond to the characteristic frequencies of absorption in **1** and the interaction with microwaves is determined by dielectric losses solely.

Obtained data allows to calculate the refraction index of the studied material:


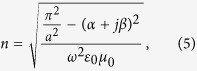


where *a* is the waveguide width and ω is the circular frequency.

As expected, there is a slight decrease of refraction index with temperature (Fig. 4b) in the region where no spin transition is observed which is caused by the thermal expansion of the complex. However, reaching the transition temperature, an abrupt increase of the refraction index is observed with SCO induced change of refraction index estimated at Δn/n ~ 6%. This cannot be explained *via* the density change related to SCO (d_LS_ = 1.98 g cm^−3^, d_HS_ = 1.778 g cm^−3^)^37^, which decreases about 11% during the transition from LS to HS states, whereas with SCO the refraction index of **1** increases.

The same abrupt increase of refraction index upon spin transition was previously observed in THz region by Mounaix *et al*.^30^. However, in case of refraction index variation investigated in visible region, only its slight decrease was observed^32^,^40^,^41^, that can be explained by thermal expansion.

As well, an increase of refraction index indicates an increase of permittivity upon SCO at 37 GHz. However, previous measurements at 1 kHz^31^ showed an abrupt drop of dielectric permittivity as a result of spin transition. Therefore, direction of the dielectric parameters variation upon spin transition strictly depends on the frequency of radiation. In general, modifications of dielectric parameters originate from the deformation of electronic molecular orbitals upon spin transition that leads to the change of metal-ligand bond length and, therefore, different electrical dipoles in LS and HS states.

In addition, due to the hysteresis of spin transition, dielectric properties of the complex are different in heating and cooling modes that causes a memory effect (in this case – an ability to possess different values of refractive index in the same conditions depending on how this state has been reached).

In conclusion, here we offer a new way of microwave switching by means of molecular spin crossover phenomenon. Due to the huge diversity of SCO complexes, this approach towards SCO microwave switches with any desired temperature, abruptness and hysteresis of spin transition can be envisaged. The change of refraction index in the microwave region that follows spin transition can make these materials employed in different microwave setups. At the same time, parallel determination of permittivity and permeability will be the topic of our further study. SCO complexes containing heavy metal ions (e.g., analogues of Hofmann clathrates^42^) can display a higher microwave absorption that makes them more attractive as functional materials for microwave devices. Chiral SCO materials^21^,^43^ also cause interest in regard to the known effect of chirality on the interaction with a microwave^44^. Because spin transition occurs on the nanosecond scale^45^, microwave switches based on SCO compounds can become a new route towards ultrafast modulation of microwave radiation.

## Methods

### Synthesis of 1

Powder sample of [Fe(trz)(Htrz)_2_](BF_4_) **1** was obtained as previously described by Kroeber *et al*.^33^. Two distinct solutions were prepared: (a) Iron (II) tetrafluoroborate hexahydrate (1.1 g, 3.3 mmol, 1 equiv) in water (7 ml); (b) 1,2,4-1H-triazole (0.69 g, 10 mmol, 3 equiv) in ethanol (3.3 ml). The two solutions were rapidly mixed and a white precipitate appeared; after a while its colour became pink. The mixture was allowed to stand for 24 h, then the complex was filtered off, washed with ethanol and dried on air. Anal. Calcd. for FeN_9_C_6_H_8_BF_4_ (%): C 20.63, H 2.29, N 36.10; found: C 20.61, H 2.37, N 36.21. IR (Nujol): 522, 634, 678, 724, 868, 978, 1070, 1162, 1308, 1496, 1534, 1748, 2536, 2700, 3174 (Figure S2). PXRD patterns are given in Figure S3.

### Magnetic studies

Temperature-dependent magnetic susceptibility measurements were carried out with a Quantum-Design MPMS-XL-5 SQUID magnetometer equipped with a 5 T magnet over the temperature range 310–385 K with a cooling and heating rate of 2 K min^−1^, and magnetic field of 0.5 T. Diamagnetic correction for the molecule was applied.

### Mössbauer spectrum measurements

Mössbauer spectrum was recorded with a ^57^Co source embedded in a rhodium matrix using a Wissel Mössbauer spectrometer. Isomer shifts are given relatively to iron metal at ambient temperature. Simulations of the experimental data were performed with Recoil software.

### Elemental analyses and IR

Elemental analyses (C, H, N) were performed with a Vario EL III element analyser. Infrared spectra were recorded with a Perkin-Elmer spectrometer BX II (4000–400 cm^−1^) in Nujol.

### Microwave attenuation studies

Microwave attenuation measurements were carried out with P2–65 scalar microwave network analyser operating in Ka frequency band. Analyser was equipped with hollow rectangular waveguides (7.2 × 3.4 mm).

### Refraction index and absorption measurements

Refraction and absorption measurements were carried out using microwave generator G4-115 equipped with measuring slotted line R1-31 based on rectangular waveguide (7.2 × 3.4 mm).

## Additional Information

**How to cite this article**: Kucheriv, O. I. *et al*. Spin-Crossover Materials towards Microwave Radiation Switches. *Sci. Rep.* **6**, 38334; doi: 10.1038/srep38334 (2016).

**Publisher’s note:** Springer Nature remains neutral with regard to jurisdictional claims in published maps and institutional affiliations.

## Supplementary Material

Supplementary Information

## Figures and Tables

**Figure 1:**
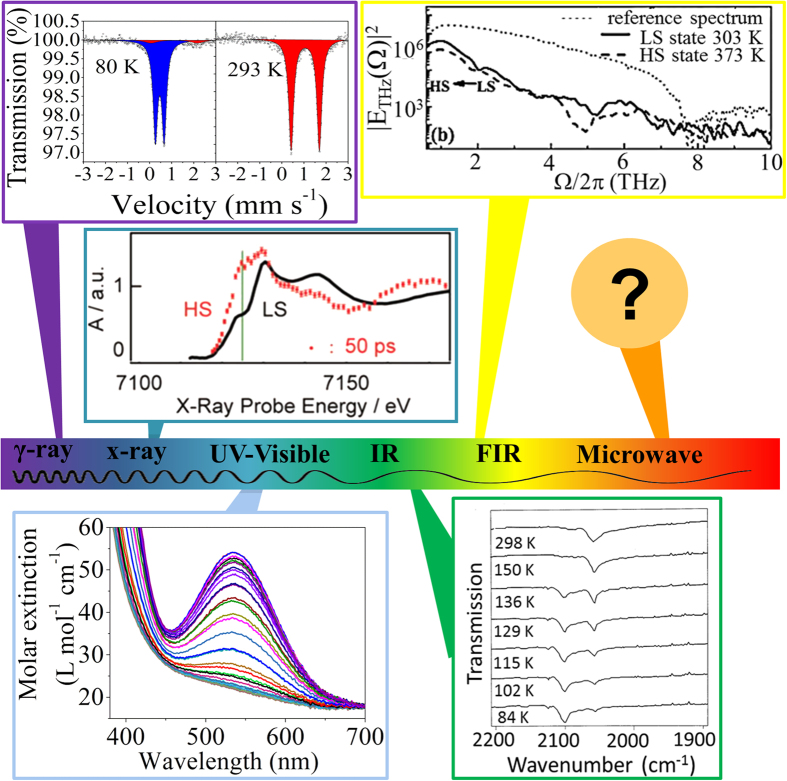
Interaction of electromagnetic radiation of different frequencies with SCO compounds. Images are reproduced from refs 39, 43, 46, 47, 48 with permission. Copyrights 2011 AIP; 2016 ACS; 2009 AAAS; 2000 Elsevier.
