# Supplementary material for: Spin-Crossover Materials towards Microwave Radiation Switches
Source: Sci Rep. 2016 Dec 2;6:38334. doi: 10.1038/srep38334 (PMC5133552; doi:10.1038/srep38334)
Supplement: Supplementary Information [file srep38334-s1.pdf]

Spin-Crossover Materials towards Microwave Radiation Switches

Olesia I. Kucheriv,[a] Viktor V. Oliynyk,[b] Volodymyr V. Zagorodnii,[b] Vilen L. Launetz,[b] and Il’ya A. Gural’skiy[a]*

Supporting information

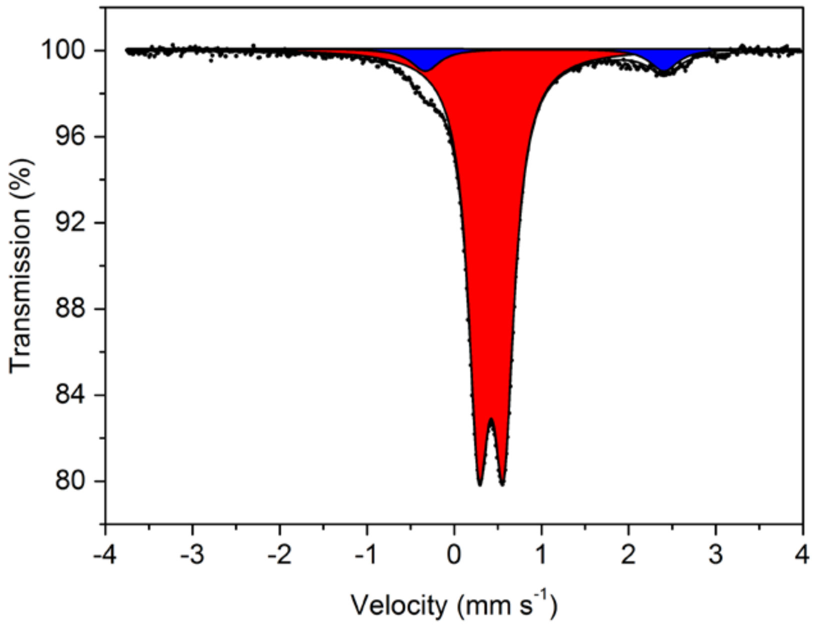

Figure S1. Mössbauer spectrum of **1** recorded at room temperature.

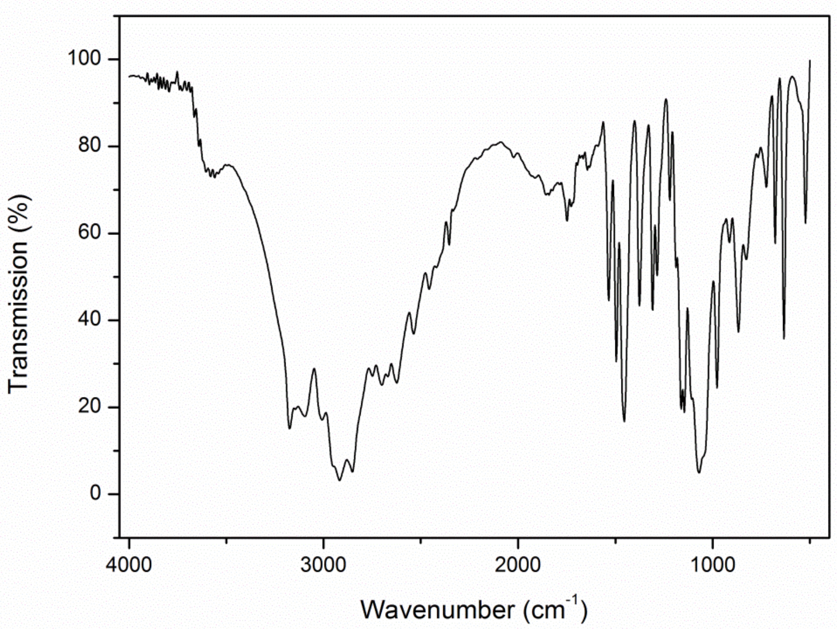

Figure S2. IR spectrum of **1** recorded in Nuiol.

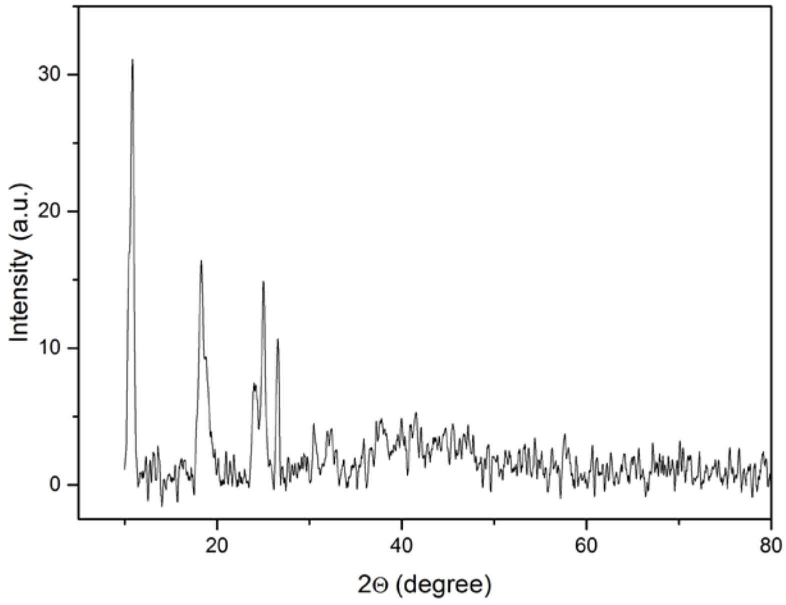

Figure S3. X-ray powder diffraction pattern of **1**.
